# Metabolic Signatures in Lung Cancer: Prognostic Value of Acid–Base Disruptions and Serum Indices

**DOI:** 10.3390/ijms26178231

**Published:** 2025-08-25

**Authors:** Florian Ponholzer, Marie-Christin Neuschmid, Helga Komi, Christina Bogensperger, Caecilia Ng, Herbert Maier, Paolo Lucciarini, Stefan Schneeberger, Florian Augustin

**Affiliations:** Department of Visceral, Transplant and Thoracic Surgery, Center of Operative Medicine, Medical University of Innsbruck, 6020 Innsbruck, Austria

**Keywords:** lung cancer, NSCLC, acid–base homeostasis, electrolyte disruptions, bicarbonate, hyponatremia, hypocalcemia, hypochloremia, VATS, surgical oncology, lung tumor, pH

## Abstract

One characteristic of tumor cells is the increased anaerobic metabolism through glycolysis leading to an acidic environment of the tumor. This acidity is linked to tumor progression, invasion and metastasis, besides stimulated survival pathways in the malignant cells. The aim of our analysis is to investigate the role of systemic acid–base parameters such as the pH, bicarbonate, baseexcess and lactate in lung cancer patients. Furthermore, alterations in electrolytes and hemoglobin were investigated regarding their impact on overall survival. Data of 937 non-small-cell lung cancer (NSCLC) patients, who underwent anatomic video-assisted thoracoscopic surgery (VATS) resection, was collected in a prospectively maintained database and analyzed. To minimize confounding effects and due to the retrospective study design, we decided to use data from the first arterial blood gas analysis during surgery and the most recent lab results prior to surgery. We found significant correlations between low systemic bicarbonate (<20 mEq/L) and overall survival (*p* = 0.006). Hyponatremia (<135 mmol/L) correlated with lower 5-year overall survival (*p* = 0.004) and decreased disease-free survival (*p* = 0.017). Hypochloremia (<98 mmol/L) was linked to reduced overall survival (*p* = 0.003) and hypocalcemia (<1.15 mmol/L) with worse disease-free survival (*p* = 0.015). Hemoglobin under 12 g/dL for women and 13 g/dL for men was associated with poorer outcomes (*p* < 0.001). Other acid–base parameters such as the pH (*p* = 0.563), baseexcess (BE) (*p* = 0.290) and lactate (*p* = 0.527) did not show significant differences in overall or disease-free (pH: *p* = 0.130; BE: *p* = 0.148; lactate: *p* = 0.418) survival. Systemic bicarbonate, sodium, calcium, chloride and hemoglobin levels were found as prognostic markers and possible therapeutic targets to improve overall survival. Further investigations are necessary to develop therapeutic strategies.

## 1. Introduction

Lung cancer presents the world’s leading cause of cancer-related deaths, with challenges in diagnosis and treatment and it remains a constant threat despite medical advances. While developments in therapeutic strategies have shown promise, the interplay between tumor microenvironment factors and disease progression necessitates a deeper understanding. One of these factors is the acid–base homeostasis of the tumor microenvironment [1,2].

Differences in the metabolism of cancer cells compared to healthy cells were first defined by Otto Warburg more than 90 years ago [3]. These variations were marked by the fact that cancer cells tend to perform more anaerobic metabolism even if the mitochondria of the cancer cell remain functional and sufficient oxygen is available. Compared to healthy cells there is an increased uptake of glucose in malignant cells and therefore more anaerobic metabolism. Insufficient blood supply in the tumor additionally accelerates hypoxia and increases vascular endothelial growth factor (VEGF) expression. These effects lead to an enhanced quantity of H+ and lactate as the basis for an acidic milieu. Correspondingly, the pH level in the tissue surrounding the tumor ranges from 6.5 to 6.9 [2,4,5,6].

These effects and the acidic environment stimulate further tumor progression, invasion and metastatic spread. Also, survival pathways in the tumor tissue are upregulated by lower pH ranges. In contrast, healthy cells are not able to adapt to the conditions in the same way as tumor cells. Reduced proliferation in normal cells due to those conditions creates space, which is then invaded by malignant cells. Zhou et al. (2023) [7] have recently found metabolic processes in cancer cells to produce extra H+ ions in order to maintain a stable intracellular pH. Key acidifiers in the cancer cell are nucleotide biosynthesis and the deployment of sialic acid. Metabolic reprogramming occurs during tumor progression; these findings provide new possibilities for diagnostics, prognostics and therapeutics [7,8]. Recent analyses show that neutralizing low pH levels in the microenvironment can inhibit this formation of metastases [4,6,7,8,9].

Under the assumption that variations in the pH of the immediate tumor environment also affect the systemic acid–base homeostasis, Sebastian et al. [10] compared and analyzed the serum bicarbonate of patients who underwent stereotactic body radiation therapy (SBRT) due to non-small-cell lung cancer (NSCLC). This study has shown a link between lower levels in serum bicarbonate (<26 mEq/L) and higher tumor recurrence [10].

As the pH level is in part influenceable, the question arises whether one could use it as a new treatment strategy. In a mouse model by Robey et al. (2009) [4], the influence of orally administered bicarbonate was investigated for prostate and breast cancer. Treated mice showed a positive effect on the incidence of metastases and increased survival [4,11].

Shie et al. (2023) [12] have shown that a more acidic tumor environment leads to enhanced vasculogenic mimicry and metastasis in a mouse model, and have therefore shown that chronic acidity is not just a by-product but it actively drives key cancer behaviors. They also found an increased production of ITGA4 integrin, which helps the tumor cell to interact with the surrounding matrix [12].

This study aimed to analyze the effect of acid–base homeostasis, electrolytes and hemoglobin levels in patients with NSCLC. The analyzed parameters (pH, bicarbonate, baseexcess, lactate, sodium, potassium, calcium, chloride, hemoglobin) are investigated as possible prognostic factors.

## 2. Results

### 2.1. Patient Demographics

Overall, 937 patients were included in the analysis, 448 (47.8%) were of female sex and 489 (52.2%) male. All UICC stages are represented in the database, with a majority of patients being staged with early-stage UICC I (60.8%) and II (21.1%) lung cancers. The rate of neoadjuvantly treated patients is comparably low at 9.7%. Patient demographics are shown in Table 1.

### 2.2. Effect of Acid–Base and Serum Indices on 5-Year Overall and Disease-Free Survival

**Figure 1:**
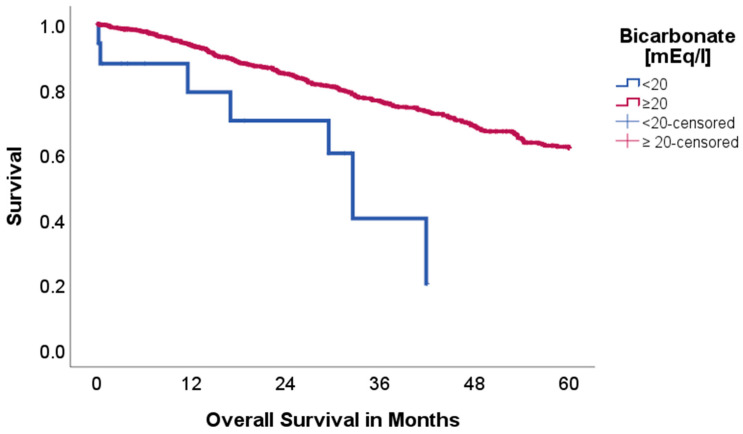
Kaplan–Meier curves with regard to overall survival stratified for bicarbonate levels.

Bicarbonate (HCO_3_):

The patient cohort with hydrogencarbonate (HCO_3_) levels under 20 mmol/L (*n* = 18 vs. 914) showed a significantly reduced 5-year overall survival (OS) with 31.7% in comparison to 62.3% (*p* = 0.006, Chi-Square: 7.413), as is visualized in Figure 1. Disease-free survival (DFS) did not differ between cohorts (83.1 vs. 63.0%, *p* = 0.577, Chi-Square: 0.312). Patients with HCO_3_ levels under 20 mmol/L had a significantly higher rate of BE under −2 mmol/L (100.0 vs. 9.0%, *p* < 0.001). A confounder analysis including age, length of stay, BMI, age-adjusted Charlson Comorbidity Index and pathological staging showed no difference between groups.

**Figure 2:**
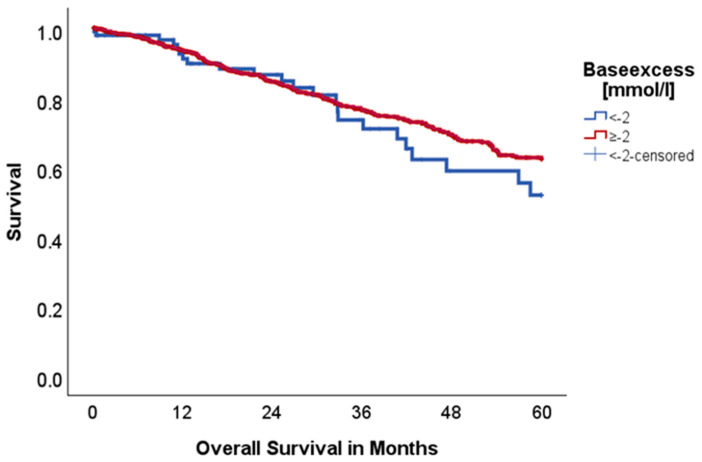
Kaplan–Meier curves with regard to overall survival stratified for baseexcess.

Baseexcess:

BE under −2 mmol/L (*n* = 100 vs. 830) did not seem to influence 5-year OS (52.1 vs. 62.5%, *p* = 0.336, Chi-Square: 0.924), although there is a graphical trend identifiable that indicates an advantage for the group with BE ≥ −2 mmol/L, as shown in Figure 2. Also, DFS did not differ between cohorts (74.8 vs. 61.6%, *p* = 0.156, Chi-Square: 2.008).

Lactate:

Lactate levels under 20.0 mg/dL (*n* = 913 vs. 22) showed no association with 5-year OS (62.0 vs. 65.2%, *p* = 0.663, Chi-Square: 0.190). DFS did not differ between cohorts (63.2 vs. 59.4%, *p* = 0.779, Chi-Square: 0.079). Lactate levels showed no association with tumor diameter (*p* = 0.359).

**Figure 3:**
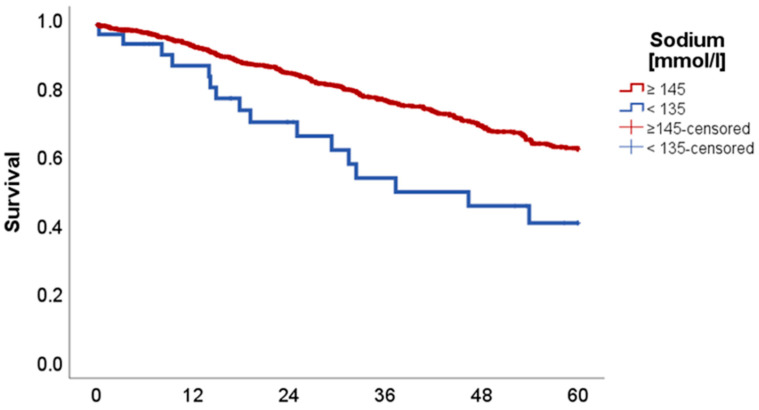
Kaplan–Meier curves with regard to overall survival stratified for sodium levels.

Sodium:

Low sodium levels (<135 mmol/L) (*n* = 35 vs. 902) showed a significant association with reduced 5-year OS with 40.8 in comparison to 62.8% (*p* = 0.004, Chi-Square: 8.224), as can be seen in Figure 3.

DFS was also significantly reduced in the cohort with sodium levels under 135 mmol/L with 49.4% in comparison to 63.5% (*p* = 0.017, Chi-Square: 5.681).

Potassium:

Abnormal potassium levels (hypokalemia: *n* = 108; normal: *n* = 935; hyperkalemia: *n* = 2) showed no association with 5-year OS and DFS (67.4 vs. 60.9% and 61.5 vs. 63.4%, *p* = 0.213/0.794, Chi-Square: 1.551/0.068).

**Figure 4:**
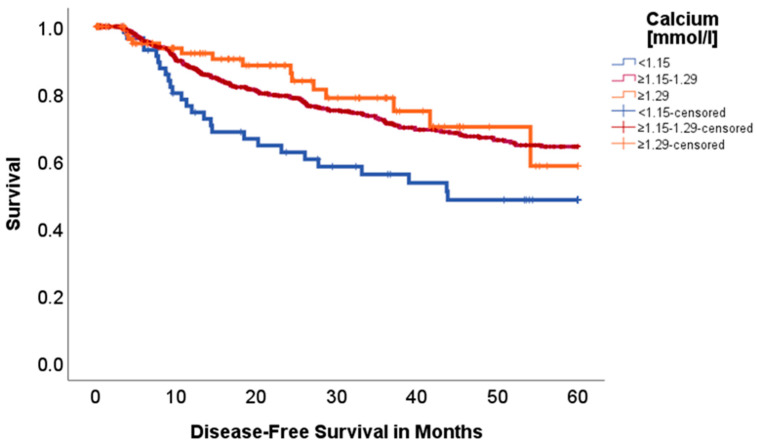
Kaplan–Meier curves with regard to disease-free survival stratified for calcium levels.

Calcium:

While hypercalcemia did not seem to make a difference, we found significantly worse disease-free survival for patients with hypocalcemia (<1.15 mmol/L, *n* = 62 vs. 776 vs. 98) (*p* = 0.015, Chi-Square: 8.365), as seen in Figure 4 (48.3% vs. 64.2% vs. 58.2%). In a subgroup analysis with low calcium vs. normal or elevated serum calcium, we saw the same effect (*p* < 0.005, Chi-Square: 7.877). No difference was found for 5-year OS (*p* = 0.526, Chi-Square: 1.286).

**Figure 5:**
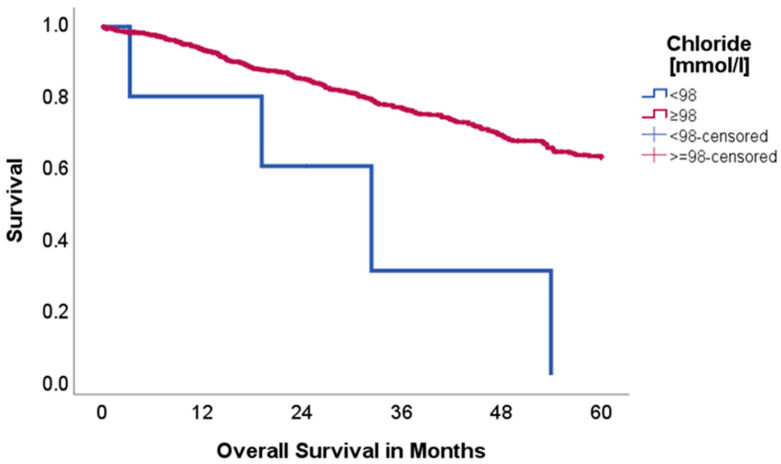
Kaplan–Meier curves with regard to overall survival stratified for chloride levels.

Chloride:

Patients with reduced chloride (<98 mmol/L, *n* = 6 vs. 930) showed significantly worse OS than patients with normal or elevated chloride levels (62.5 vs. 0.0%, *p* = 0.003, Chi-Square: 8.990), as is visualized in Figure 5.

Chloride levels appeared to have no significant impact on disease-free survival (*p* = 0.189, Chi-Square: 1.723).

**Figure 6:**
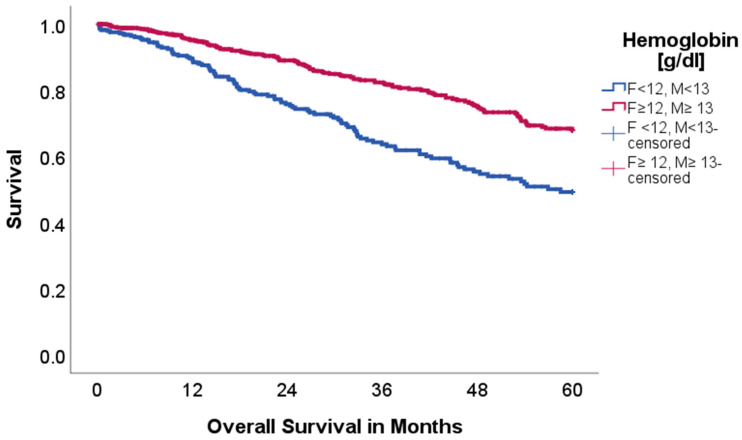
Kaplan–Meier curves with regard to overall survival stratified for hemoglobin levels.

Hemoglobin:

Low hemoglobin levels (adjusted for gender) (*n* = 287 vs. 650) were significantly associated with reduced 5-year OS with 50.0% in comparison to 68.2% (*p* < 0.001, Chi-Square: 24.736); visualized in Figure 6.

DFS was comparable between cohorts, with a graphical trend towards reduced DFS in the low hemoglobin cohort (63.9 vs. 62.6%, *p* = 0.745, Chi-Square: 0.106).

To assess for a possible confounding effect of neoadjuvant therapy on low-hemoglobin patients, the survival analysis was also performed without patients who received neoadjuvant therapy. Still, the low-hemoglobin cohort showed reduced 5-year OS with 52.8 in comparison to 70.2% (*p* < 0.001).

DFS did not differ, when excluding patients after neoadjuvant treatment (67.1 vs. 63.8%, *p* = 0.603).

## 3. Materials and Methods

### 3.1. Patient Selection and Data Collection

Data of patients who underwent video-assisted thoracoscopic surgery (VATS) anatomic resections as treatment for primary lung cancer between February 2009 and December 2021 at the Department of Visceral, Transplant and Thoracic Surgery (Medical University of Innsbruck) was collected in a prospectively maintained database. Exclusion parameters were defined as metastases from other cancers, benign pathologies and insufficient laboratory parameters. After enforcing the exclusion parameters, data of 937 from 953 patients was further analyzed.

The study was conducted in accordance with the Declaration of Helsinki (as revised in 2013). Permission was granted by the local ethics committee. Because of the retrospective study design, no additional blood samples could be taken from patients. To minimize confounding effects, we analyzed the first arterial blood gas analysis at the day of surgery and serum parameters from the most recent lab results prior to surgery.

The limits and ranges for laboratory values (Table 2) were the used limits of the central institute for medical and chemical laboratory diagnostics (ZIMCL) of the Tirol Kliniken GesmbH.

### 3.2. Statistics

Statistical analyses were performed using IBM Statistics SPSS 29 (IBM Corporation, Armonk, NY, USA) and the R software environment with the ‘survminer’ and ‘ggplot2’ package to plot survival curves [15,16]. Student’s *t*-test was used for the comparison of two means. The Kaplan–Meier estimator in combination with the log-rank test was used to analyze overall and disease-free survival. Statistical significance was assumed for *p*-values < 0.05.

## 4. Conclusions

This study analyzed possible associations of acid–base and serum parameters with the outcome for surgically resected NSCLC patients. Since cancer cells possess an increased anaerobic metabolism, even if sufficient oxygen is available, excess acidic derivates are built. This leads to an increasingly acidic tumor environment, which may cause advancing tumor aggressiveness and metastasis [4,5,6,9]. The aim of this study was to retrospectively explore this possible association of acid–base and serum parameters with the oncological outcome.

Data from Sebastian et al. (2019) [10] has shown that the pH in the immediate tumor environment seems to be linked to the systemic acid–base homeostasis. As a possible result, the rate of tumor recurrence was negatively correlated with serum bicarbonate (<26 mEq/L) [10]. Robey et al. showed in a mouse model that orally administered HCO_3_ increases the extracellular tumor pH and reduces the amount of metastasis in metastatic breast cancer. Furthermore, reduced lymph node involvement was seen [4]. If tumor recurrence and serum bicarbonate levels are truly connected, this might offer a well-tolerated therapy at low cost to improve oncological outcomes. In our cohort a serum bicarbonate cut-off of <26 mEq/L did not show an impact on OS and DFS. Nevertheless, levels <20 mEq/L showed a significant association with reduced 5-year OS (31.7 vs. 62.3%, *p* = 0.006), but no association with DFS (83.1 vs. 63.0%, *p* = 0.577). The exact reason for this reduced OS, but not DFS, may be due to further underlying diseases rather than the oncological interactions with the tumor microenvironment prior to surgery. Although serum bicarbonate plays a key role in acid–base homeostasis and 100% of patients with HCO_3_ levels under 20 mmol/L had a BE under −2 mmol/L, BE did not show an association with OS and DFS.

Changes in lactate metabolism may cause lactic acidosis and hyperlactatemia, which contributes to further acidosis [17]. Lung cancer cells are intense consumers of glucose, but glucose levels in the tumor itself tend to be low and lead to increased anaerobic metabolism with the associated rise in lactate levels. Even if glucose is sufficiently available, cancer cells still tend to keep high anaerobic metabolism levels [18]. As a possible parameter of a more acidic metabolism and/or increased lactate production, which usually occurs in more aggressive and larger tumors, lactate levels were also analyzed, but interestingly showed no association with OS and DFS. Furthermore, lactate levels were no indicator of tumor size (*p* = 0.359). Interestingly, even when comparing more aggressive G3 tumors with G1 tumors, no association between lactate levels and tumor grading was found (8.97 vs. 9.40, *p* = 0.358). As a result, it may be hypothesized that larger and/or more aggressive tumors do not produce more and enough lactate to actually increase systemic lactate levels than their smaller and/or less aggressive counterparts.

Electrolyte disorders are a well-recognized phenomenon in oncology, often reflecting the underlying disease state, general condition of the patient and treatment-related effects. Beyond sodium level disturbances, our analysis revealed notable findings regarding disbalances in calcium and chloride homeostasis. Sodium levels and associated hyponatremia have been systemically linked to prolonged hospitalization, reduced performance status of patients and a negative impact on oncological outcomes. The incidence in the available literature ranges from 3.0 to 94.8% in lung cancer patients, with higher rates in small-cell lung cancer according to most of the literature. Still, the impact of hyponatremia seems to be more severe in patients with NSCLC. The exact pathophysiology behind hyponatremia in lung cancer and its negative impact is still not fully understood, but data shows that it is mostly linked to the syndrome of inappropriate antidiuretic hormone secretion [19,20]. Also, in our cohort, patients with sodium levels <135 mmol/L showed significantly worse OS with 40.8% (vs. 62.8%, *p* = 0.004). In contrast to the above-mentioned parameters, low sodium levels were additionally associated with reduced DFS (*p* = 0.017). The question remains whether these low sodium levels are in regard to the underlying oncological disease or should be seen as completely independent. Nevertheless, it is a condition that should be tackled, as a meta-analysis by Corona et al. has shown reduced mortality in hyponatremic patients if their serum sodium levels are improved [21].

While hypercalcemia is a well-studied metabolic complication of malignancy, hypocalcemia remains rather underrepresented. Nevertheless, recent studies claim that hypocalcemia occurs in up to 11% of lung cancer patients [22,23,24]. To further distinguish our results, a direct measurement of ionized calcium via blood gas analysis was performed, avoiding the need for mathematical corrections typically required for total serum calcium and therefore avoiding possible bias. Ionized calcium represents the physiologically active fraction, making it a more accurate indicator of calcium status [25]. Interestingly, we found a significant advantage for patients with normal or elevated calcium blood levels regarding DFS (*p* = 0.015). The clinical manifestations of hypocalcemia vary widely, ranging from neuromuscular irritability and tetany to fatigue and depressive symptoms. In severe cases, hypocalcemia can contribute to cardiac arrhythmias, including QT prolongation [26]. Still, the etiology of hypocalcemia in lung cancer patients is multifactorial and not clearly investigated yet. Electrolyte imbalances and acid–base disorders often serve as a marker for systemic issues rather than being directly attributable to the tumor itself. Potential contributors include adverse side effects of medication, like chemotherapeutic-induced metabolic alterations. Platinum-based chemotherapeutics, as widely used in lung cancer, are known for their nephrotoxic effects and can subsequently affect renal calcium handling [24]. However, our data did not demonstrate a significant correlation between calcium levels and renal function (*p* = 0.404). Although hypocalcemia is often associated with bone metastases [23], this does not appear to apply in our cohort, as none of the patients with hypocalcemia presented with bone metastases. Although alterations in potassium are often seen in cancer patients [24], we did not find any correlation of hypo- or hyperkalemia regarding DFS and OS (*p* = 0.794; *p* = 0.213).

Furthermore, chloride levels were also shown to be a prognostic marker for our patient population. Hypochloremia can be observed in up to 23% of lung cancer patients [24] and was associated with worse OS outcomes in our cohort (*p* = 0.003). The exact mechanisms underlying this association remain unclear. In summary, our findings highlight the complex interplay between electrolyte imbalances and lung cancer progression. Further research is necessary to reveal the prognostic significance of these disturbances and their potential role in guiding clinical management strategies.

In this study we further analyzed hemoglobin levels as a biomarker for oncological outcomes. Several studies already described hemoglobin as a possible factor for dismal prognosis in lung cancer patients [27,28]. Low hemoglobin levels are a common finding in cancer patients, but there is no clarity regarding the underlying mechanisms. A likely hypothesis is that tumors produce various substances, such as tumor necrosis factor- α or interleukin−6, which might impact hematopoiesis. This might even be aggravated through bone marrow metastases. As a result, the patient and its tumor are exposed to lower oxygen levels and possible hypoxia. This may in turn increase tumor aggressiveness and chemotherapy resistance through the above-mentioned pathways [29]. In our cohort low hemoglobin levels were also associated with reduced OS (*p* < 0.001), but not reduced DFS (*p* = 0.745), which might speak against the hypothesis of increased tumor aggressiveness through anemia. Even when adjusting for a neoadjuvant chemotherapy, low hemoglobin levels were a significant risk factor for reduced OS (*p* < 0.001), but again not DFS (*p* = 0.603).

Considering our results, we can provide four possible biomarkers for reduced OS following surgical resection of lung cancer. All of these parameters can be easily measured and in many cases also corrected. Knowledge of the impact of those parameters can provide an easy and economical target to improve patient survival without impacting their quality of life. Nevertheless, to prove our described impact, prospective trials investigating the therapeutic use of HCO_3_, electrolytes and hemoglobin correction need to be performed.

Limitations:

Due to the retrospective characteristics of this study, further serum parameters were not available for analysis. It has to be taken into consideration that in our analysis, patients are usually preoxygenated before intubation, which might impact various analyzed parameters in our study. Preoxygenation is known to increase pCO2 and pO2 and therefore decreases pH, while standard bicarbonate remains unaffected [30]. The increase in pCO2 can lead to respiratory acidosis but only affects serum bicarbonate when it requires renal acidification mechanisms [31,32]. Further, significant electrolyte shifts are uncommon during preoxygenation and initial anesthesia. Nonetheless, some anesthetic agents (e.g., halothane) have been associated with minor alterations in serum sodium or calcium, but only when used over a prolonged period [33].

## Figures and Tables

**Table 1:** Patient demographics.

Factor	n = 937	%
** Sex **		
Females	448	47.8
Males	489	52.2
Mean Age [y] (median, IQR *)	64.34 (65.0, 14)	
** Mean BMI [kg/m^2^] (median, IQR) **	25.38 (24.9, 5.77)	
** Mean Length of Stay [days] (median, IQR) **	10.24 (8.0, 5)	
Age-Adjusted Charlson Comorbidity Index **		
0	47	5.0
1	83	8.9
2	196	20.9
3	233	24.9
4	169	18.0
5	102	10.9
≥6	107	11.4
** pT **		
0	12	1.3
1	559	59.7
2	254	27.1
3	97	10.4
4	15	1.6
** pN **		
0	683	73.3
1	134	14.3
2	115	12.3
** pM **		
0	911	97.5
1	22	2.3
2	1	0.1
** UICC **		
I	570	60.8
II	198	21.1
III	135	14.4
IV	25	2.7
** Pathology **		
Adeno	657	70.1
Squamous	161	17.2
Neuroendocrine	74	7.9
Mixed	17	1.8
Large-Cell	11	1.2
Small-Cell Lung Cancer	9	1.0
Others	8	0.9
Clavien Dindo ***		
<III	824	90.4
≥III	87	9.6
** Neoadjuvant therapy **	91	9.7

* Interquartile range. ** Age-adjusted Charlson Comorbidity Index is used to classify comorbidities, as described by Charlson et al. (1987) [13]. *** Clavien Dindo was used to grade postoperative complications as described by Dindo et al. (2004) [14]. Missing patient data is not separately reported.

**Table 2:** Limits and ranges for laboratory values.

Analyte [ASTRUP]		Range/Limit
Hemoglobin		
	Female	12.0–15.7 g/dL
	Male	13.0–17.7 g/dL
Bicarbonate		20.0–26.0 mmol/L
Baseexcess (BE)		≥−2–+3 mmol/L
Sodium		136–146 mmol/L
Calcium^2+^ (ionized)		1.15–1.29 mmol/L
Chloride		98–106 mmol/L
Lactate		4–20 mg/dL

## Data Availability

The data presented in this study are available on request from corresponding author. The data are not publicly available due to privacy reasons.
